# Transcriptomic Analysis Reveals Differential Gene Expressions for Cell Growth and Functional Secondary Metabolites in Induced Autotetraploid of Chinese Woad (*Isatis indigotica* Fort.)

**DOI:** 10.1371/journal.pone.0116392

**Published:** 2015-03-04

**Authors:** Yingying Zhou, Lei Kang, Shiying Liao, Qi Pan, Xianhong Ge, Zaiyun Li

**Affiliations:** National Key Lab of Crop Genetic Improvement, National Center of Crop Molecular Breeding Technology, National Center of Oil Crop Improvement (Wuhan), College of Plant Science and Technology, Huazhong Agricultural University, Wuhan, P. R. China; Institute of Genetics and Developmental Biology, Chinese Academy of Sciences, CHINA

## Abstract

The giant organs and enhanced concentrations of secondary metabolites realized by autopolyploidy are attractive for breeding the respective medicinal and agricultural plants and studying the genetic mechanisms. The traditional medicinal plant Chinese woad (*Isatis indigotica* Fort., 2*n* = 2*x* = 14) is now still largely used for the diseases caused by bacteria and viruses in China. In this study, its autopolyploids (3*x*, 4*x*) were produced and characterized together with the 2*x* donor for their phenotype and transcriptomic alterations by using high-throughput RNA sequencing. With the increase of genome dosage, the giantism in cells and organs was obvious and the photosynthetic rate was higher. The 4*x* plants showed predominantly the normal meiotic chromosome pairing (bivalents and quadrivalents) and equal segregation and then produced the majority of 4*x* progeny. The total 70136 All-unigenes were *de novo* assembled, and 56,482 (80.53%) unigenes were annotated based on BLASTx searches of the public databases. From pair-wise comparisons between transcriptomic data of 2*x*, 3*x*, 4*x* plants, 1856 (2.65%)(2*x* vs 4*x*), 693(0.98%)(2*x* vs 3*x*), 1045(1.48%)(3*x* vs 4*x*) unigenes were detected to differentially expressed genes (DEGs), including both up- and down-regulated ones. These DEGs were mainly involved in cell growth (synthesis of expansin and pectin), cell wall organization, secondary metabolite biosynthesis, response to stress and photosynthetic pathways. The up-regulation of some DEGs for metabolic pathways of functional compounds in the induced autotetraploids substantiates the promising new type of this medicinal plant with the increased biomass and targeted metabolites.

## Introduction

Polyploidy which contributed greatly to the evolution of angiosperms is involved in the speciation of many important crops, such as the autopolyploids alfalfa and potato or more frequent allopolyploids bread wheat, cotton, oilseed rape, coffee, etc. [1, 2]. Allo- and autopolyploids have genetically been distinguished by modes of chromosome pairing and inheritance, for allopolyploids exhibit bivalent pairing and disomic inheritance but autopolyploids show multivalent pairing and polysomic inheritance [3]. Induced polyploids have long been used as useful tools to study the morphological modifications and the underlying mechanism in polyploids, because the autopolyploidy is usually associated with the increased size in plant architecture, organs and cells [4, 5, 6]. The results from tetraploidizing various *Arabidopsis thaliana* mutants and transgenics with a wide range in cell size indicated that the ploidy-dependent increase in cell volume is genetically regulated [6]. Early study reported that the chloroplast number and photosynthesis per cell all increase with ploidy increase, which were attributed to increased size of cells [7]. But the recent experiment showed that not the nuclear ploidy but the cell area was the key parameter determining the activity of chloroplast proliferation, while the expression of the related genes was not promoted in the lines with the defect in cell proliferation but enhanced post-mitotic cell expansion [8]. So the mechanisms behind the ploidy-related regulation of cell size, cell proliferation and expansion, cellular proliferation remain largely for further studies [8].

For the popular occurrence of polyploidy, particularly allopolyploidy, the extensive investigations of genetic consequences of genome mergers have been made in diverse plant taxa during last 20 years and the dynamic nature of polyploid genomes and widespread changes to gene expression as revealed by transcriptomic analysis [9, 10, 11, 12, 13, 14]. The results showed that the genome merger rather than genome doubling per se was responsible for considerable transcriptomic alterations in natural and synthetic allopolyploids [14, 15]. On the contrary, the several studies on the gene expression of autopolyploid plants failed to detect significant transcriptomic alterations as found in allopolyploids, and only showed that autopolyploid experienced narrow reorganization of gene expression. The comparative analysis of 9,000 genes revealed very weak differences between potato diploid and autopolyploid [3]. Similarly, nearly 4.3% of all probe sets showed differences between the diploid and autotetraploid *Isatis indigotica*, by using the *Arabidopsis thaliana* whole genome genechip [16]. Recently, autopolyploidy was reported to cause increased cytosine methylation, besides enhancing accumulation of secondary metabolites in the aromatic *Cymbopogon* grasses [17]. Particularly, the transcriptome alterations in *Arabidopsis thaliana* autotetraploids produced from different ecotypes strongly depended on their parental genome composition and include changed expression of both new genes and gene groups found in its derived allopolyploid [5]. Furthermore, alterations in gene expression were stable, nonstochastic, developmentally specific, and associated with changes in DNA methylation [5].

However, despite the morphological and physiological advantages associated with the plant autopolyploidization, the artificially synthesized autopolyploids by doubling the chromosome number of donor diploids often suffered from the cytological unstability and the chromosome variations among progenies, because the existence of four copies of each chromosome caused the formation of multivalents besides bivalents, then the chromosome missegregation and finally the gametes of variable chromosomes and low fertility. These meiotic aberrations are commonplace in most newly formed autopolyploid plants [18, 19], which hindered the subsequent utilization of the induced autopolyploids as commercial crops with the aim of harvesting seeds, while the seedless autotriploid watermelon was preferred by the customers.

*Isatis indigotica* Fort. (Chinese woad, 2*n* = 14), a member of *Isatideae* tribe of the Brassicaceae family [20], is a biennial herbaceous plant which has been used as an important and popular medicinal plant with a long history in China and also likely as indigo-producing plant in ancient times. The medicine made from its dried roots (*Radix Isatidis*) can be used for antibacterial, antiviral, and immune regulatory effects in the treatment of colds, fever, and influenza [21]. Additionally, this plant is also utilized as valuable germplasm for resistance breeding of *Brassica* crops [22], because it shows resistance to tobacco mosaic virus (TMV) [23] and stem rot (*Sclerotinia sclerotiorum*) [24], the most serious disease of rapeseed in China. The synthetic autotetraploid *Isatis indigotica* was shown to have higher content of active compounds which were regarded as its effective constituent [25]. However, in spite of its large amount usage, the study of genetics and molecular biology for this plant is very limited.

No report about the genome size of Chinese woad was found. But another closely related species *Isatis tinctoria* L. (Woad, 2n = 28) in the *Isatideae* tribe which is mainly distributed in Europe has the double chromosome number and 0.58pg 1C DNA amount [20]. The genome of Chinese woad was sequenced by State Key Laboratory of Dao-di Herbs, China Academy of Chinese Medical Sciences, but the heterozygosity of the line used hindered assembly of high quality (personal comm.). But hundreds of its SSR markers were developed and used to identify individual chromosomes in alien background [26]. The genome size was estimated to be ~300Mb, equaling to about two times of *Arabidopsis thaliana* [27], but to nearly one half of the cultivated *Brassica* diploids which have the genome size of ~600Mb [28]. So its chromosomes are of smaller size, even compared with those of *Brassica* species with small chromosomes [20, 22, 26]. The small genome of the Chinese woad is likely suitable for genome manipulation through inducing the autotetraploid, in order to breed the new type with larger roots and higher content of medicinal compositions, as reported for it and other species [17]. In this study, we explored the phenotypic and physiological changes by the comparison of diploid and synthetic autopolyploid. A comprehensive survey of global gene expression in response to ploidy levels was performed for its diploid, autotriploid and autotetraploid by using Illumina RNA-Seq, to elucidate the gene expressions related to the changes in phenotype, cell size, physiology and functional secondary metabolites. The results gave some new insights into the genetic regulation associated with the plant autopolyploidization.

## Materials and Methods

### Plant materials

*Isatis indigotica* Fort. (2*n* = 2*x* = 14) (provided by Jiangsu Germplasm Repository) was used as the diploid donor. Surface-sterilizing of the seeds were proceeded using a water solution with ethanol at a concentration of 70% for 1–2 min and in 0.1% HgCl_2_ (w/v) for 15 min, then rinsed with sterile water for three times. Then the seeds were germinated on MS medium [29] with hormone free. After germination, the seedlings were cultured at 25°C ±3°C under the white fluorescent light with a photoperiod at about 16h. For artificially synthesizing the autotetraploids, the plantlets were transferred into MS agar medium supplemented with 1.5 mg L^-1^ 6-BA, 0.25 mg L^-1^ NAA and 100 mg L^-1^ colchicine and grown for 2 weeks, and then they were transferred to MS medium without colchicine to generate shoots. Then the rooted plantlets (S_0_: synthesized autotetroploid) were transplanted to the experimental fields in our university. Triploid was produced via crossing between diploid and tetraploid plants, and S_0_ was self-pollinated to generate S_1_. The diplod, triploid and tetraploid S_1_ plants which were planted in the greenhouse were used for Illumina RNA-Seq.

### Flow cytometric analysis

The supposed diploid, triploid and tetraploid (S_0_, S_1_) plants were sampled for ploidy level analysis via Quanta SC Flow Cytometer (Beckman Coulter, USA). The method was almost the same as previously described [30] with some modifications. 100 mg fully developed leaf tissue were chopped in 400 μl ice-cold nuclear isolation buffer (15 mM NaCl, 50 mM glucose, 15 mM KCl, 50 mM sodium citrate, 5 mM Na_2_EDTA, 50 mM HEPES, 0.5% (v/v) Tween 20, 0.5% (v/v) β-ME, pH = 7.2) to obtain the nuclei suspensions. Then the nuclei suspension was filtered through a 50μm nylon filter and stained with 200 μl ice-cold DAPI staining solution (4′-6-diamidino-2-phenylindole, 0.4 mg/ml). Then the samples were analyzed for ploidy level by a flow cytometer. Before analyzed by the flow cytometer, all processes must operate on ice. Leaves from diploid plants were used as control.

### Cytological and pollen viability analysis

To determine the chromosome numbers of synthetic plants, the ovaries from young flower buds were collected and treated with 2 mM 8-hydroxyquinoline for 3–4 h at room temperature before fixed in Carnoy’s solution I (3:1 ethanol: glacial acetic acid, *v*/*v*) and stored at -20°C for further study [31]. Pollen fertility was determined as the percentage of pollen grains stained with 1% acetocarmine, and more than 300 pollen grains from three flowers of each plant were stained [31], also comparison of the pollen size between S_0_ and diploid was conducted. The cytological images were captured with a CCD camera attached to the fluorescence microscope (Nikon Eclipse 80i). Images were processed by Adobe Photoshop (Adobe Systems, San Jose, CA) to adjust contrast and brightness.

### Photosynthesis investigation

Photosynthetic rates were measured in one mature leaf with a portable photosynthesis system (LI-6400XT, LI-COR, USA). The leaves of the plantlets grow under the same environment and each of the samples has three repetitions. When measuring photosynthesis, the photosynthetic photon flux density was set with 1000 μmol m^-2^s^-1^ and cuvette block temperature was 24°C, and concentration of the CO_2_ was set at 350 μmol mol^-1^ with a flow rate of 500 ml s^-1^. The concentrations of CO_2_ were controlled by a buffer bottle. All of the measurements were carried out from 9:00 to 11:00 in sunshine weather. The chamber was attached to a leaflet, the photosynthesis allowed to stabilize and the data recorded.

### RNA extraction, library preparation, Illumina sequencing

The fourth newly expanded leaves from two plants of each of diploid, triploid and tetraploid were collected as one sample at seedling stage, and two biological replicates were made. As the young plants of autoploids (3*x*, 4*x*) grew some lower than those of diploids, we chose to collect the leaves samples at the same node, as other researcher did [6], though these leaves were not at the exactly the same developmental stage, because the autoploid leaves grew some slower. Otherwise, if we sampled the leaves after certain duration of seed sowing, it was more difficult to define the development stage. The young plants of three ploidy levels did not show very obvious difference in growing rate at early stage. Total RNA of each sample was isolated with TRIzol reagent according to the manufacturer’s instructions (Invitrogen, USA). RNA integrity was verified by 1.5% Agrose gel electrophoresis and confirmed using a 2100 Bioanalyzer analyzer (Agilent, CA, USA). The mRNA enrichment, RNA fragmentation, the first and second strand cDNA synthesis and purfying, sequencing adaptors ligation and PCR amplification were performed as previously described [32]. For high-throughput sequencing, the libraries were applied to Illumina sequencing platform (HiSeq 2000, SanDiego, CA, USA) using a paired-end read protocol with 100 bp of data collected per run.

### Data processing and *de novo* assembly

After sequencing, the raw image data was transformed into sequence data by base calling, which was saved as fastq format and named raw reads. The raw reads were quality filtered using filter-fq to remove reads with adaptors, reads containing more than 5% of unknown nucleotides, and low quality reads. Transcriptome *de novo* assembly is carried out with short reads assembling program-Trinity within each sample [33]. Unigenes from each sample’s assembly were taken into further process of sequence splicing and redundancy removing to acquire non-redundant unigenes as long as possible by TGICL [34]. Then do gene family clustering, the unigenes will be divided to two classes. One is cluster, which the prefix is CL and the cluster id is behind, the other are singletons, which the prefix is Unigene.

### Functional annotation

Blastx alignment (E-value < 0.00001) between unigenes and protein databases like Nr (NCBI non-redundant database), Nt (NCBI non-redundant nucleotide database), Swiss-Prot (Swiss-Prot protein database), KEGG (Kyoto Encyclopedia Of Genes and Genomes) and COG (Clusters of Orthologous Groups of proteins) is performed as previously described [35]. When a unigene happens to be unaligned to any of the above databases, ESTscan [36] was used to predict its direction. With Nr annotation, Blast2GO program was used to get GO annotation of unigenes. After getting GO annotation for each unigene, we used WEGO software to do GO functional classification for all unigenes and to understand the distribution of gene functions of the species from the macro level [37]. KEGG pathway annotation is performed using Path_finder software against the KEGG database.

### Analysis of differentially expressed genes (DEGs) and GO and pathway enrichment

The reads of the diploid, triploid and tetraploid samples were mapped back to our *de novo* assembling results using RSEM [38]. To evaluate the gene expression, the number of unique-match reads was calculated and then normalized to FPKM (Fragments per Kilo base of transcript per Million mapped reads) which was used to calculate the unigene expression with the restrictive conditions of | log2Ratio | ≥1.0 and FDR≤0.001. Then the results were submitted to Path_finder and Blast2GO for enrichment analysis. GO enrichment analysis of these DEGs was performed using blast2GO with P-value≤1 and pathway enrichment analysis used Path_finder software against the KEGG database with Q-value≤1.

## Results

### Synthesis and phenotype of woad autopolyploids (3*x*, 4*x*)

**Fig 1:**
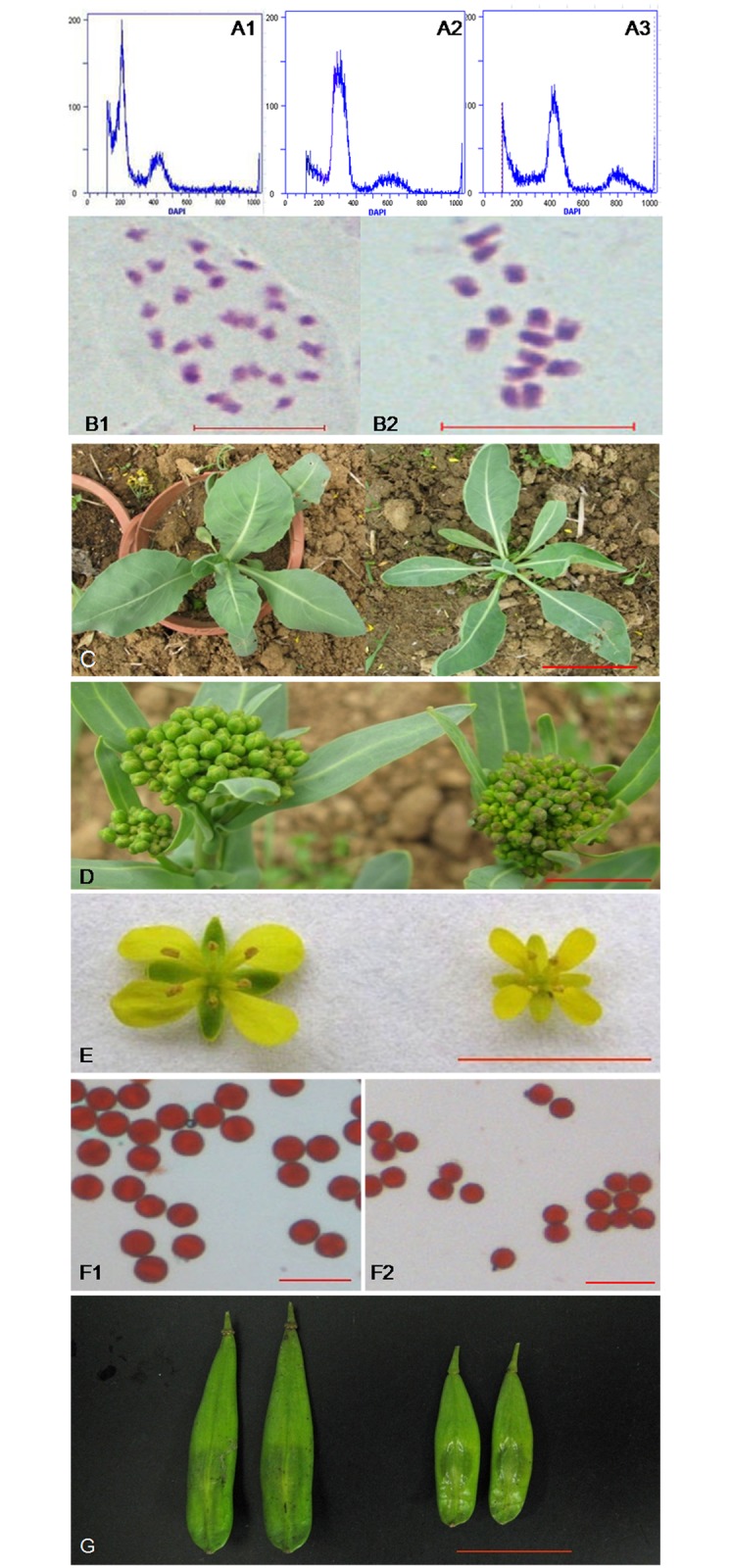
Ploidy analysis and phenotypic characterization of autopolyploid. A: Ploidy analysis of 2*x* (A1), 3*x* (A2), 4*x* (A3) woad by flow cytometer. B: Mitotic metaphase of 4*x* plant with 28 chromosomes (B1) and 2*x* plant with 14 chromosomes (B2). C-G: Morphological differences between diploid and autotetraploid, C-G: plantlets, inflorescence, flowers, pollen grains and siliques of autotetraploid (left) and diploid (right). Scale bars: B, F = 10 μm; C = 10 cm; D, E, G = 1 cm.

After the colchicine treatment of diploid donor on MS medium, 25.4% plantlets regenerated were identified to have the doubled chromosome number (2*n* = 4*x* = 28), as revealed by flow cytometric and cytological analysis (Fig. 1). The doubled plants (S_0_) produced the majority of 4*x* S_1_ progeny by self-pollination of plants, resulting from the high frequency formation of bivalents and quadrivalents and equal segregation of chromosomes during the meiotic divisions of pollen mother cells (S1 Table). Triploid plants were obtained from the 4*x* × 2*x* cross with the tetraploid as female and confirmed via flow cytometric analysis (Fig. 1).

**Fig 2:**
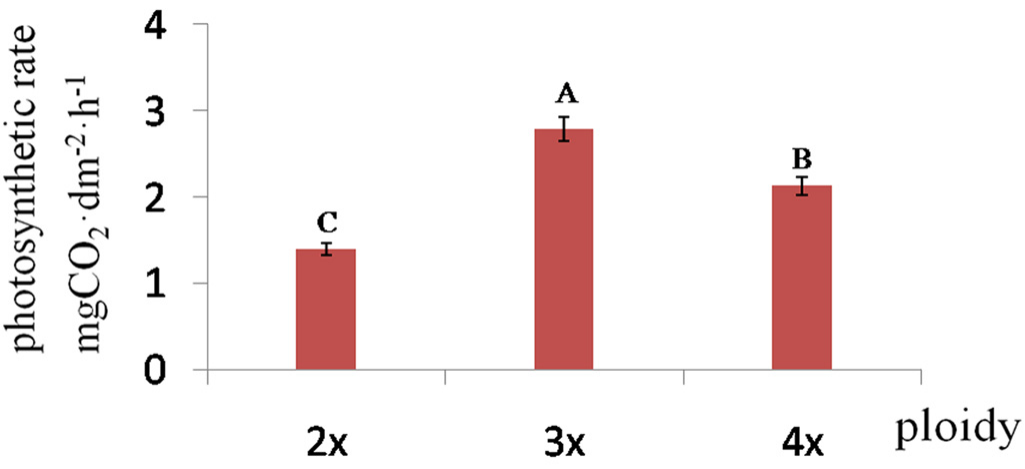
Photosynthetic rate of woad with different ploidy. A, B, C revealed that the photosynthetic rate were significantly different (P<0.01) among 2*x*, 3*x* and 4*x* plants.

The 4*x* woad plants showed morphological variations compared with diploid donor, such as slower plant growth, darker green pigmentation, thicker, wider and larger leaves, as often observed amongst the colchicine-treated populations (Fig. 1) [4, 39, 40]. The plants of larger size produced more branches, larger flowers and pods (Fig. 1). They also produced the pollen grains of larger size, a significant feature of autopolyploidy (Fig. 1) [40, 41]. The pollen stainability of the tetraploid was very high (95.9%), nearly the same as diploid donor (96.8%), which was responsible for the good seed-set. The morphology and size of leaves and plant architecture of the triploid plants were more similar to that of diploid donor than to the tetraploid, though some difference was detectable. Interestingly, the triploid exhibited the highest net photosynthetic rate (2.79±0.18 mg CO_2_·dm^-2^·h^-1^), followed by the tetraploid (2.13±0.06), while both were much higher than the diploid (1.39±0.04) (Fig. 2).

### Overview of Illumina RNA sequence and *de novo* assembly

To obtain an overview of the woad transcriptome at 2*x*, 3*x*, 4*x* levels, cDNA libraries was generated from leaves of young plants, then Illumina paired-end sequencing was conducted. From the total 92548258 reads produced from six samples, 87446592 clean reads (94.5%) were obtained with an average length of 90 bp (Table 1) after cleaning and quality checks (number of reads per sample was described in S2 Table and these data set is available in NCBI’s Gene Expression Omnibus (http://www.ncbi.nlm.nih.gov/geo/) under accession number GSE61103). Then the cleaned reads from six samples were *de novo* assembled separately using the short-read assembly program Trinity to yield unigenes. These unigenes were further clustered into 70,136 All-unigenes (hereinafter referred to as unigenes) with a mean size of 1068 bp, including 29,049 clusters and 41,087 singletons. There were 29,597 unigenes (42.20%) with a length range from 200 to 500 bp, 31,044 unigenes (44.26%) longer than 1000 bp and no unigenes shorter than 200 bp. Distinct Singletons contained some high similar (more than 70%) unigenes, and these unigenes might come from same genes or homologous genes.

**Table 1:** Illumina RNA-Seq reads and *de novo* assembly statistics of woad.

Total number of raw reads	92548258
Total number of clean reads	87446592
Mean length of reads (bp)	90
Total number of unigenes	70136
Mean length of unigenes (bp)	1068
Total length(bp)	74919265
N50	1816

### Functional annotations and encrichement of the unigenes from woad leaf

**Fig 3:**
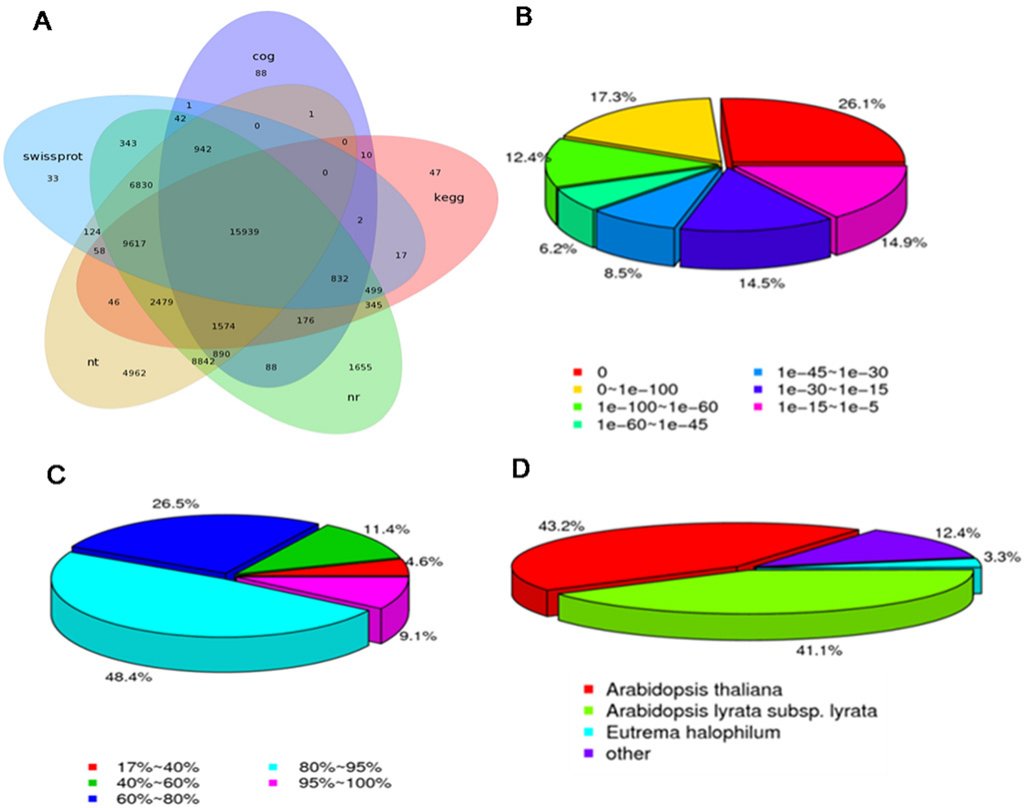
Unigenes annotatation and characteristics of homology search of unigenes against the nr database. A: Venn diagram of number of unigenes annotated by BLASTx with an E-value threshold of 10^-5^ against the 5 databases. B: E-value distribution of the top BLAST hits against the nr database for each unique sequence. C: Similarity distribution of the top BLAST hits against the nr database for each unique sequence. D: Species distribution of unigenes in the nr database.

A total of 56,482 (80.53%) unigenes were annotated based on BLASTx (cut-off E-value 10^-5^) searches of the public databases: Nr, Nt, Swiss-Prot, KEGG and COG (S3 Table). Among these unigenes, 51,093 (72.8%) unigenes could be annotated with reference to the nr database and 15,939 (22.73%) were annotated by all five databases (Fig. 3A).

Based on Nr annotation and the E-value distribution, 55.8% of the mapped sequences shared very strong homology (E-value <10^-60^) and 14.7% had strong homology (10^-60^< E-value <10^-30^), and 23% showed homology (10^-30^< E-value <10^-5^) (Fig. 3B). The similarity distribution was depicted in Fig. 3C, among these sequences, 57.5% had similarities higher than 80%, while 42.5% of the hits had similarities of 17–80%. As regards to species distribution, 43.2% of the distinct sequences had top matches to sequences from *Arabidopsis thaliana*, followed by *Arabidopsis lyrata* (41.1%) and *Eutrema halophilum* (3.3%) (Fig. 3D).

**Fig 4:**
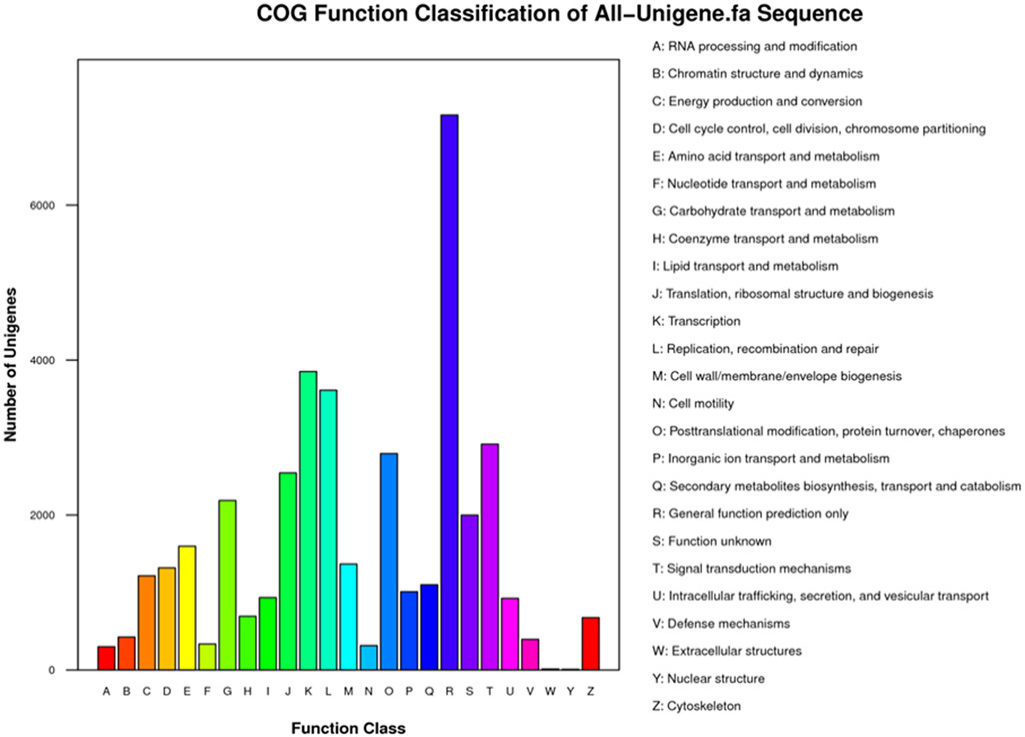
COG function classification. All the unigenes aligned in COG database were assorted in 25 clusters.

There were 20,585 unigenes annotated by COG databases and assigned to 25 COG function clusters (Fig. 4). Among the 25 clusters, the “general function prediction only” cluster comprised the highest number of unigenes (7162, 34.79%), the “Transcription” and the “Replication, recombination and repair” cluster had the second (3852, 18.71%) and the third (3610, 17.54%) largest number of unigenes. By contrast, only 8 unigenes were classified into “nuclear structure”.

**Fig 5:**
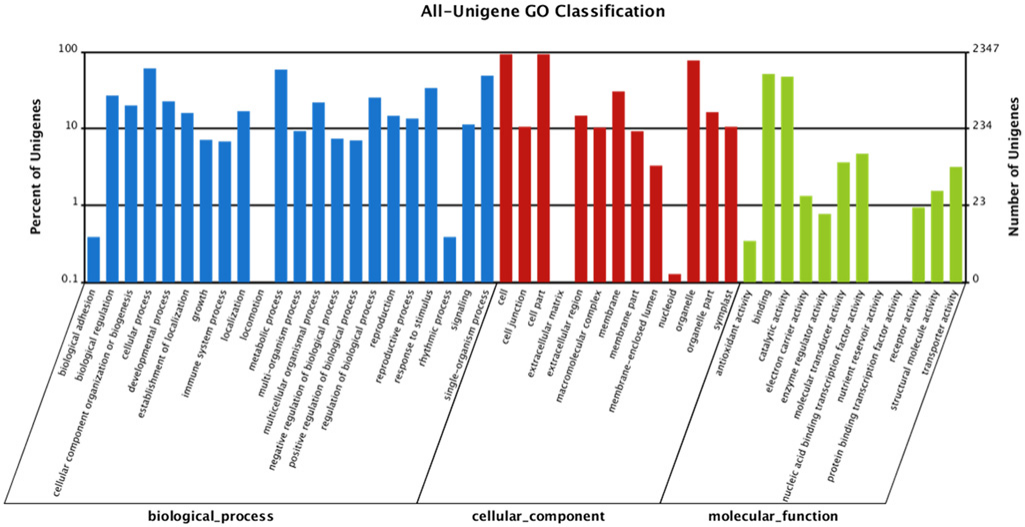
Classification of GO annotations. The x-axis indicates the sub-categories; the left y-axis indicates the percentage of a sub-category of genes in that category and the right y-axis indicates the number of unigenes in a sub-category.

GO (Gene ontology) annotation of unigenes was obtained by using Blast2GO program with Nr annotation. 45,150 unigenes were classified into 47 groups which could be categorized into three main classifications: “biological process”, “cellular component” and “molecular function” (Fig. 5). For the biological process category, cellular process (31,237 unigenes) and metabolic process (29,695 unigenes) represented the major proportion. In the “cellular component” classification, 41,347 unigenes were involved in the “cell” and “cell part”. For the “molecular function”, binding had the maximum number of unigenes: 23,720.

There were 31,641 unigenes were annotated based on the KEGG database and mapped into 128 KEGG pathways (S4 Table). The number of unigenes in different KEGG pathways ranged from 2 to 6825. The maps with highest unigene representation were Metabolic pathways (Ko01100, 6825 unigenes, 21.57%), followed by Biosynthesis of secondary metabolites (Ko01110, 3633 unigenes, 11.48%) and Plant-pathogen interaction (Ko04626, 2237 unigenes, 7.07%).

### GO and pathway analysis of DEGs between 2*x*, 3*x* and 4*x* woad

**Fig 6:**
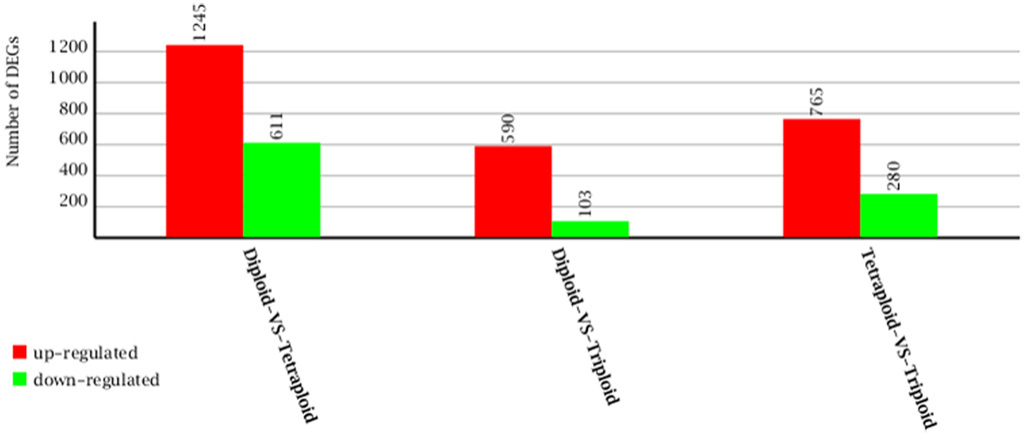
Analysis of differently expressed genes via pairwise contrasts of different ploidy with | log2Ratio | ≥ 1.0 and FDR ≤ 0.001.

Differences in gene expression of the three samples at different ploidy levels were examined. The reads of the 2*x*, 3*x*, 4*x* samples were mapped back to our *de novo* assembling results separately using RSEM. As a result, 1856, 693 and 1045 unigenes showed differential expression including both up-regulated and down-regulated unigenes between 2*x* and 4*x*, 2*x* and 3*x*, 3*x* and 4*x*, respectively (Fig. 6).

GO enrichment analysis of DEGs was performed based on the Nr database using blast2GO. For the 2*x* vs 4*x* couple, 864 DEGs were annotated and classified into 38 categories with corrected P-value≤1, involving metabolism, growth, development, catabolism, reproduction and signaling, etc. Between 3*x* and 4*x*, 280 DEGs were classified into 34 categories, and 459 DEGs into 35 categories for 3*x* and 4*x* woad (S1 Fig.).

As to the pathway differences between ploidy levels (2*x* vs 4*x*, 2*x* vs 3*x*, 3*x* vs 4*x*), a total of 108, 79 and 101 different metabolic pathways were found (Q-value ≤1), respectively (S1 File). In the pathway of 2*x* vs 4*x* DEGs, biosynthesis of metabolic pathways (152, 25.8%) had the highest number of unigenes and the most represented pathways were secondary metabolites pathway (94, 15.9%), photosynthesis (3, 0.51%), plant-pathogen interaction (58, 9.8%) and plant hormone signal transduction (30, 5.08%). The most represented pathways for 2*x* vs 3*x* and 3*x* vs 4*x* DEGs were almost the same as those of 2*x* vs 4*x* (S1 File).

### DEGs related with ploidy-specific characters

Among DEGs between 2*x* and 4*x*, three “indoleacetic acid metabolic and biosynthetic process” related genes were detected with two up-regulated and one down-regulated (Table 2). Also, “phenylpropanoid / terpenoid / flavonoid metabolic and biosynthetic process” genes were detected and most of them were up-regulated (Table 2). Five signal transduction genes (Calcium-dependent protein kinase and Receptor-like kinase) were identified. One hundred and thirteen unigenes were clustered into a GO term of “response to stimulus”, of which 63 (55.8%) unigenes were up-regulated and 63 unigenes were clustered into “response to stress” with 35 (55.6%) up-regulated, indicating that *Isatis indigotica* tetraploids were more responsive and adaptable to stresses than the diploid progenitor [42]. In addition, some unigenes related with “cell wall and cell wall organization or biogenesis”, “cell growth” and “cell cycle” were identified and most of them were up-regulated in 4*x* plants (Table 2).

**Table 2:** Functional classification of some differentially expressed genes (DEGs) in diploid vs tetraploid.

Functional characterization	GeneID	Nr-ID	log2Ratio	Q-value
Signal transduction				
CDPK	CL4413.Contig2_All	gi|334186798	-2.84705	0.802662
	CL2151.Contig1_All	gi|334186798	3.984985	0.816485
	CL4413.Contig1_All	gi|334186798	12.58232	0.929986
Receptor-like kinase	Unigene6578_All	gi|16040952	12.02143	0.897157
Receptor-like protein	CL2541.Contig1_All	gi|15221162	-2.961529	0.832724
Secondary metabolism biosynthetic				
indoleacetic acid biosynthetic and metabolic process	Unigene19992_All	gi|297828279	-11.162359	0.802099
	CL4935.Contig2_All	gi|255579783	11.464035	0.843939
	CL2978.Contig1_All	gi|297831632	14.663510	0.983354
phenylpropanoid/flavonoid biosynthetic and metabolic process	CL969.Contig6_All	gi|15219988	4.0063495	0.835619
	Unigene11987_All	gi|27311623	12.554157	0.928603
	Unigene12989_All	gi|297829892	5.9880542	0.837822
	CL843.Contig1_All	gi|297853242	11.280277	0.819836
terpenoid biosynthetic and metabolic process	CL2651.Contig2_All	gi|15219789	11.155355	0.801229
	CL4190.Contig3_All	gi|297831748	5.4094017	0.801001
Celluar				
cell growth	Unigene2323_All	gi|297795677	11.688315	0.86862
	Unigene1759_All	gi|15233283	4.0986459	0.824862
	CL84.Contig2_All	gi|22328885	5.3958078	0.853214
	Unigene7054_All	gi|1655830	6.0299786	0.84657
cell cycle	Unigene25790_All	gi|297802414	-11.358321	0.829945
	CL4062.Contig1_All	gi|30688234	12.297905	0.914972
	Unigene29128_All	gi|42567412	-11.464188	0.843939
	CL4651.Contig1_All	gi|297838807	11.951521	0.891568
	Unigene15644_All	gi|297836828	11.245136	0.814667
	CL1793.Contig2_All	gi|15218276	12.649368	0.933294
cell wall	Unigene1055_All	gi|1521920|	2.2376180	0.810311
	CL2111.Contig1_All	gi|15231618	2.6303426	0.82633
	CL5415.Contig3_All	gi|297820490	-2.934915	0.812037
	CL5415.Contig2_All	gi|297820490	-12.508265	0.926521
	Unigene11372_All	gi|259451	2.488059	0.825482
	CL649.Contig5_All	gi|75309020	2.806054	0.819158
cell wall organization or biogenesis	CL7223.Contig2_All	gi|297827065	11.561980	0.855131
	Unigene1759_All	gi|15233283	4.098645	0.824862
	CL6239.Contig1_All	gi|297801570	7.009774	0.852308
	Unigene33219_All	gi|449439966	11.444885	0.841843

The 280 differentially expressed genes were annotated by comparing triploid with diploid. Genes related to “response to stress” and “response to stimulus” were identified and with 91.3% (21/23) and 91.7% (33/36) up-regulated, respectively, and one CDPK gene (CL4413.Contig1_All) was found. Nine “growth”, “cell cycle” and “cell wall” related genes were detected and all of them had the increased expression.

With regard to GO cluster of 4*x* vs 3*x* DEGs, 3 “indoleacetic acid metabolic and biosynthetic process” related genes were detected with 2 up-regulated, while genes related to “phenylpropanoid/ terpenoid/ flavonoid metabolic and biosynthetic process” were absent. Some “response to stimulus and stress” related genes were detected with almost 82% up-regulated and obviously triploid plants had higher expression of “response genes” than diploid and tetraploid plants. The expression of many “growth and cell growth” related genes all increased. With genes related to photosynthesis, there were some genes differentially expressed in the comparison of three couples and most of the DEGs were up-regulated (Table 3), which was consistent with their different photosynthetic rates.

**Table 3:** Functional classification of some differentially expressed genes (DEGs) related in photosynthesis via KEGG pathway enrichment analysis.

pathway	2n vs 4n DEGs		2n vs 3n DEGs		4n vs 3n DEGs	
	GeneID	Nr-ID	GeneID	Nr-ID	GeneID	Nr-ID
Photosynthesis-antenna proteins	CL1451.Contig1_All↑^a^	gi|297815030	CL1451.Contig1_All↑	gi|297815030	CL3215.Contig1_All↑	gi|297829418
	CL1451.Contig2_All↑	gi|312281813	CL1451.Contig5_All↑	gi|297815028		
	CL1451.Contig5_All↑	gi|312281813				
	CL1451.Contig4_All↑	gi|4741948				
Carbon fixation in photosynthetic organisms	CL3895.Contig2_All↑	gi|21537361	Unigene22621_All ↑	gi|1362025	Unigene24627_All↑	gi|56112311
	Unigene1954_All ↑	gi|297830044	Unigene38902_All ↑	gi|226509134	Unigene38902_All ↑	gi|226509134
			Unigene17704_All ↑	gi|470115774	Unigene3183_All↓^b^	gi|406727
			Unigene4708_All ↑	gi|312283141	Unigene12511_All↑	gi|452823767
			Unigene1954_All ↑	gi|297830044	CL987.Contig1_All ↓	gi|297801782
			Unigene20039_All ↑	gi|325185409	Unigene20039_All ↑	gi|325185409
Photosynthesis	CL3652.Contig1_All↓	gi|297813905			Unigene3609_All ↑	gi|112030956
	CL3652.Contig2_All↑	gi|297813905				
	CL6588.Contig2_All↑	gi|83032226				

Pathways with Q-value ≤ 1 are significantly enriched in DEGs.

^a^ “↑” represents up-regulated.

^b^ “↓” represents down-regulated.

Intersection of differentially expressed genes of 2*x* vs 4*x*, 2*x* vs 3*x* and 4*x* vs 2*x* was carried out and only two unigenes were detected (Unigene14441_All and CL2541.Contig1_All). Unigene14441_All (gi|297819604), which was a hypothetical protein ARALYDRAFT_485314 clustered into GO terms of “cellular process” and “organelle or plastid organization”, was up-regulated in pairwise contrasts of the three samples and had the highest expression quantity in the 3*x* plant. CL2541.Contig1_All (gi|15221162) was a receptor like protein 15 (RLP15) which was involved in signal transduction. The expression of CL2541.Contig1_All decreased in 3*x* and 4*x* plants when compared with 2*x* plant and 3*x* had the lowest expression of the RPL15. This indicated that these two DEGs were not regulated by DNA content and there might be some DEGs up-regulated or down-regulated along with the increase of DNA content whereas they were not significantly different. One of the “cell growth” unigenes, Unigene1759_All (AT3G55500) encoding the protein expansin, showed the mean FPKM values of 5, 17, 85 in 2*x*, 3*x*, 4*x* plants, respectively, but the expression difference was not significant between 2*x* and 3*x*, or 3*x* and 4*x*, but only significant between 2*x* and 4*x*.

## Discussion

From the study on the induced polyploidy plants, some important characteristics such as enhanced biomass yield [43] and resistance to both drought and low temperatures can be achieved through chromosome doubling. Then the larger size of the floral organs, delays in flowering, prolongations of the flowering period, larger fruits and greater yield production [44, 45] make polyploids also of great interest to both agriculture and horticulture. Similarly, the synthesized 4*x* woad expressed the architecture giantism (Fig. 1), as shown by other plant types. The knowledge that the polyploidy related giantism was attributed to the ploidy-dependent cell enlargement, not to the more cell number has been obtained and widely accepted for fungi, plants, and animals [2, 4, 5, 6, 46, 47]. But the molecular mechanisms for how the cell volume is regulated by the ploidy level remain largely elusive [48]. The hypothesis that increases in gene copy number increase the amount of protein, which in turn increases the cell volume seemed not to explain the phenomenon, because the relationship between the cell size and the amount of protein would not be linear. As we know, the cell structure is composed of linear structures (e.g., DNA, RNA, microtubules), dimensional planes (various membrane structures), and three-dimensional structures (e.g., vacuoles, cytosol) [6]. Some data suggested that ploidy level may affect cell volume, but the magnitude of cell expansion is likely under control of genetic pathways [6].

Transcriptomic analysis of an autopolyploidy series by using microarray and more powerful Illumina RNA-Seq should reveal the genome-wide alterations in gene expressions and find out the candidate genes related to the cell enlargement, or other pathways. Such studies have been performed in synthetic autopolyploid for *Arabidopsis thaliana*, *Paulownia fortunei*, birch, Rangpur lime and *Isatis indigotica* [5,16,49,50, 51], and the rates of gene expression change varied from 1.08% in Rangpur lime [51] to 12.6% in birch [50]. In our 4*x* Chinese woad, 2.65% (1856/ 70136) unigenes were detected to be differentially expressed via using high-throughput RNA sequencing, and the rate was much lower than 6.09% in tetraploid *Paulownia* [49] and 12.6% in birch [50], also by RNA-Seq. The variable percentages of differential gene expression from pair-wise comparisons between diploid and derived autotetraploid likely originated from the different types of plant or even the genome composition of the same plant, as significant ecotype specific differences in gene expression alterations was revealed in *Arabidopsis thaliana* [5]. In our study, the expression of the 4 unigenes participating in the “cell growth” were all up-regulated in the tetraploid compared with the diploid, and all of the 5 “growth” unigenes up-regulated in the triploid (Table 2). Unigene1759_All (AT3G55500) was one of the “cell growth” unigenes and encoded a protein called expansin. Expansin was secreted by the plant cell and unlocked the network of cell wall polysaccharides, permitting turgor-driven cell enlargement [52]. The polyploidy related giantism which was attributed to the larger cell size likely resulted from these up-regulated “cell growth” unigenes. This result also suggested that the cell enlargement was mainly caused by the key factors / proteins responsible for the development of cell structure, not solely the amount of proteins.

Pectin which is exclusively localized to the primary cell wall can play a role in the formation of supporting tissue [53]. We detected some unigenes that related to “cell wall” and “cell wall biogenesis” and most of these unigenes were up-regulated (Table 2). CL2111.Contig1_All (*PME35*) was one of the “cell wall” related unigene which encoded a pectin methylesterase. Loss-of-function mutant alleles of *Arabidopsis thaliana PME35* showed a pendant stem phenotype and an increased deformation rate of the stem [54]. Up-regulated *PME35* might benefit the better anti-lodging ability in our 4*x* woad, which strengthened the expanded primary cell wall.

One of the ideal expectations for the medicinal autopolyploids was that the organ giantism was accompanied by the higher content of some chemical compositions, especially the functional compounds. It was reported that the yield of the artemisinin in tetraploid *Artemisia annua* L. was 1.5 times higher than diploid plants [39]. The active compounds identified in *Isatis indigotica* and the related species *Isatis tinctoria* [55] were mainly divided into three categories: indole alkaloids, phenylpropanoids and terpenoids [56]. The content of active compounds in the leaves and roots of autotetraploid *Isatis indigotica* was observed to be higher than the diploid [57], and some new compounds including alkaloids, phenylpropanoids and organic acids were also isolated in roots of the autotetraploid selected for many generations [58, 59, 60]. From the analysis of DEGs between the diploid and tetraploid, most of the unigenes related to indoleacetic acid/phenylpropanoid/ terpenoid/flavonoid metabolic and biosynthetic processes were up-regulated (Table 2). However, such trends were unobvious for 2*x* vs 3*x* and 3*x* vs 4*x*, except for two of three indoleacetic acid biosynthetic related unigenes being up-regulated among 3*x* vs 4*x* DEGs. This provided the genetic basis for the advantage of tetraploid Chinese woad over the diploid partner by producing higher content of active chemicals [25, 57]. This result substantiated that the artificially synthesized woad autotetraploid promised for a new type with better quality and deserved further exploitation. The content of active compounds in the woad autotetraploid produced by us and other [57] deserved further analysis.

In plants, Ca^2+^-dependent protein kinases (CDPKs) were important sensors of Ca^2+^ flux in response to varieties of biotic and abiotic stress [61]. The expression of *CDPK* which encoded plant receptor-like kinases (RLKs) was induced in response to various environmental in different plant species [62]. *Isatis indigotica* tetraploids were reported to be more responsive and adaptable to stresses than the diploid by the changes in expression patterns of a cold inducible *CDPK1* [42]. In our study, the expression of *CDPK*s and *RLK*s tended to be higher in the tetraploid than in diploid (Table 2), which possibly made the tetraploid more adaptable under stress by altering CDPK mRNA level. Other tetraploids also exhibited an enhanced resistance and better adaptation to the environmental stress [42, 40]. So induced autotetraploid plants seemed to be more adaptable to stressful conditions through altering the gene expression related to certain pathways [61, 63].

In the present study, some unigenes encoding photosynthesis-antenna proteins, carbon fixation in photosynthetic organisms and photosynthesis proteins were differentially expressed (Table 3). In 2*x* vs 4*x* DEGs, almost all of the photosynthesis related unigenes were up-regulated, including antenna protein genes, carbon fixation genes and photosystem II subunit Q-2 genes. CL1451.Contig4_All encoded the light-harvesting chlorophyll a/b-binding (LHC) proteins which constituted the antenna system of the photosynthetic apparatus [64]. CL3895.Contig2_All (At3g22960) encoded pyruvate kinase (PK) which was a glycolytic enzyme converting phosphoenolpyruvate (PEP) into pyruvate in carbon fixation. These up-regulated photosynthetic genes may result in higher photosynthetic rate in polyploidy plants, as shown here or others [7, 65].

One major concern for the accurate detection of differentially expressed genes among serial ploidy levels of one plant was that the materials were sampled at suitable time and tissue, because the plants of higher ploidy usually tended to grow more slowly and produce the organs of larger size. Otherwise, the differences detected resulted likely from the growth retardation, not from the direct effect of genome dosage. Though it was a difficult choice between the developmental time and organ, it seemed that the newly expanded leaves from very young plants were optimal for this purpose, for they showed the limited difference of growth situation at early stage. At later stage, the flower organs or developing seeds were rational, because their development stage could be exactly defined and then the gene expressions compared with high accuracy. Anyhow, the plants responded to genome dosage within certain range by showing larger organ / cell size and growth retardation which were related each other. So a linear relationship was possible between the detected differences in the expression levels of cell growth genes for cell size and ploidy levels. For example, in this study, Unigene1759_All (AT3G55500) for expansin, showed the increase of expression levels along with the increase of ploidy levels, by giving mean FPKM values of 5, 17, 85 for 2*x*, 3*x*, 4*x* plants, respectively. The 3*x* plants had the expression level of the gene lower than the intermediate between 2*x* and 4*x* plants, which was consistant with its phenotype and growth biased to the 2*x* plants. The result also provided evidence that the detected difference between 2*x* and 4*x* was not the secondary effect of retarded growth but the direct effect of gene expression. In other side, it should be noted that the expression levels of cell growth genes were likely not proportional to the cell size and level of growth retardation, for no simple linear relationship between the cell size and the amount of protein was demonstrated [6]. In reverse situation, the excess copies of the genomes over certain range hindered but not enhanced plant growth [2, 4, 6], it should be interesting to observe the expression level of growth genes in these autoploids with abnormal phenotype. Similarly, the increase of secondary metabolites accumulated in 3*x* and 4*x* plants should not be in accordance with the expected from the RNA-Seq data. The further study of other aspects in autoploids was needed for elucidating the regulation of polyploidy on cell size and growth, particularly the reproductive organs.

In spite of small chromosome size of *Isatis indigotica*, the synthesized 4*x* plants showed predominantly the normal chromosome pairing (bivalents and quadrivalents) and then equal segregation during the meiotic divisions of pollen mother cells (PMCs) (S1 Table), and finally produced the high pollen fertility and 4*x* progeny. This also further showed that the probability of multivalent formation in autopolyploids was independent of chromosome length [66] but under control of genetic factors [67]. The newly generated *Arabidopsis thaliana* autotetraploids showed higher multivalent frequencies than the naturally established lines, in spite of their small sizes [68], which also revealed that the cytological diploidization proceeded during the autopolyploidization process. The excess of bivalent pairing over multivalents observed in our new woad autotetraploids (S1 Table) should accelerate its cytological diploidization and stabilization, which provides the genetic basis for their breeding and selection as new medicinal plant, as demonstrated by previous example of this plant [58, 59].

## Supporting Information

S1 Fig Functional categories of differentially expressed genes in the Gene Ontology. GO categories that were significantly enriched (P-value ≤ 1) were analyzed in pairwise comparisons (A: 2*x* vs 4*x*, B: 2*x* vs 3*x*, C: 4*x* vs 3*x*).
(TIF)

S1 File KEGG pathway enrichment analysis of DEGs in different ploidy levels (2x vs 4x, 2x vs 3x, 3x vs 4x) with Q-value ≤ 1. (ZIP)

S1 Table Numbers of PMCs at diakinesis and metaphase I with different pairing configurations of 4*x* woad. (DOCX)

S2 Table Number of raw and clean reads from the six samples. (XLSX)

S3 Table Unigenes annotated with each database. (XLSX)

S4 Table KEGG pathway identified in the woad transcriptome. (XLSX)
