# Supplementary figures and images for: Transcriptomic Analysis Reveals Differential Gene Expressions for Cell Growth and Functional Secondary Metabolites in Induced Autotetraploid of Chinese Woad (Isatis indigotica Fort.)
Source: PLoS One. 2015 Mar 4;10(3):e0116392. doi: 10.1371/journal.pone.0116392 (PMC4349453; doi:10.1371/journal.pone.0116392)

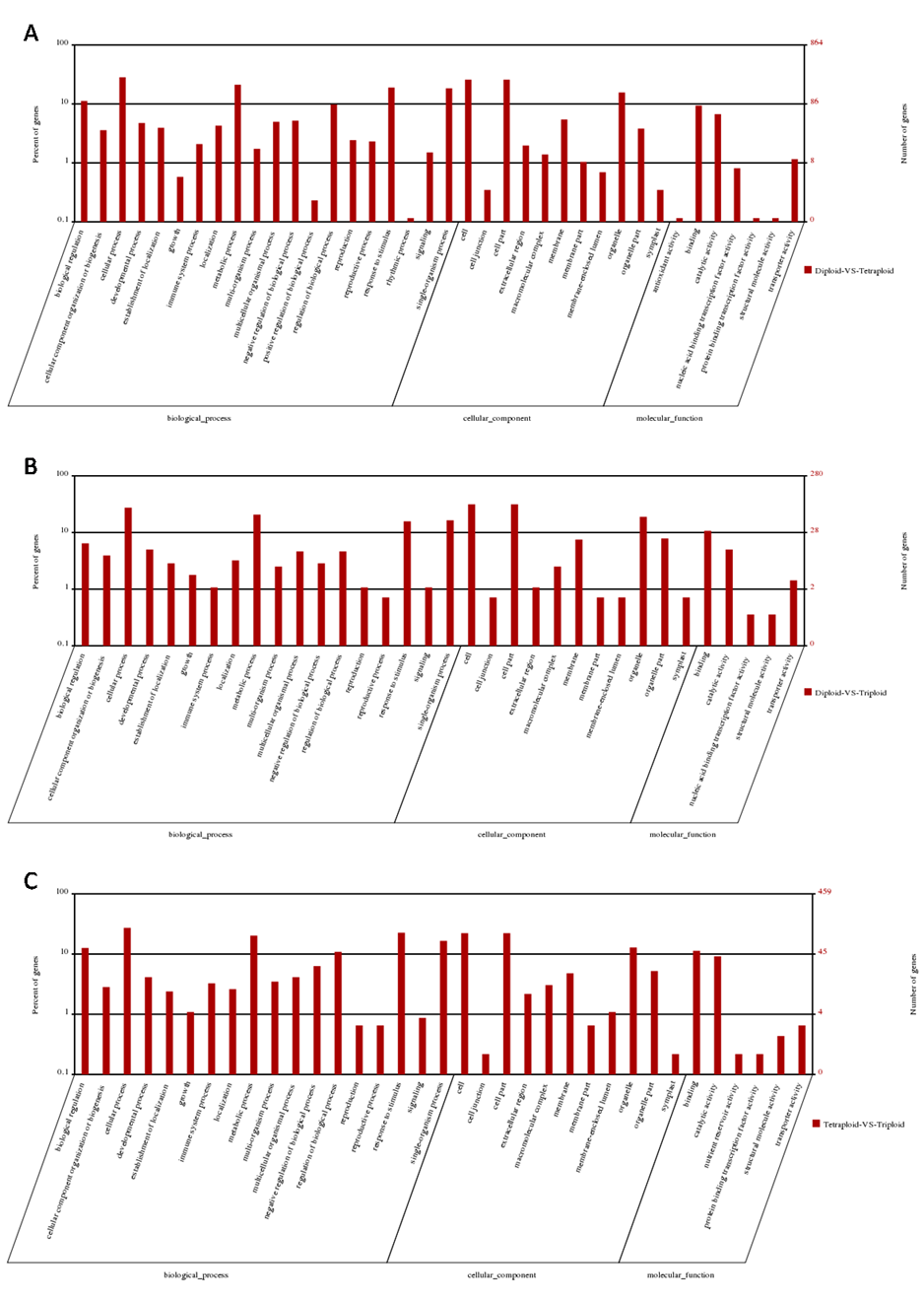

Supplement: S1 Fig — GO categories that were significantly enriched (P-value ≤ 1) were analyzed in pairwise comparisons (A: 2x vs 4x, B: 2x vs 3x, C: 4x vs 3x). (TIF) [file pone.0116392.s001.tif]
