# Supplementary material for: Transcriptomic Analysis Reveals Differential Gene Expressions for Cell Growth and Functional Secondary Metabolites in Induced Autotetraploid of Chinese Woad (Isatis indigotica Fort.)
Source: PLoS One. 2015 Mar 4;10(3):e0116392. doi: 10.1371/journal.pone.0116392 (PMC4349453; doi:10.1371/journal.pone.0116392)
Supplement: S1 Table — (DOCX) [file pone.0116392.s003.docx]

**Table S1** **Numbers of PMCs at diakinesis and metaphase I with different pairing configurations of 4*x* woad.**

| chromosome pairings | | | | | | | | | | | | | | | | |
| --- | --- | --- | --- | --- | --- | --- | --- | --- | --- | --- | --- | --- | --- | --- | --- | --- |
| 14Ⅱ |  | 12Ⅱ+1Ⅳ |  | 10Ⅱ+2Ⅳ |  | 8Ⅱ+3Ⅳ |  | 6Ⅱ+4Ⅳ |  | 4Ⅱ+5Ⅳ |  | 2Ⅱ+6Ⅳ |  | 7Ⅳ |  | total |
| 4 |  | 9 |  | 21 |  | 29 |  | 26 |  | 9 |  | 5 |  | 3 |  | 106 |
